# A Deep Learning Model for Automated Sleep Stages Classification Using PSG Signals

**DOI:** 10.3390/ijerph16040599

**Published:** 2019-02-19

**Authors:** Ozal Yildirim, Ulas Baran Baloglu, U Rajendra Acharya

**Affiliations:** 1 Department of Computer Engineering, Munzur University, Tunceli 62000, Turkey; baloglu@munzur.edu.tr; 2 Department of Electronics and Computer Engineering, Ngee Ann Polytechnic, Singapore 599489, Singapore; aru@np.edu.sg; 3 Department of Biomedical Engineering, School of Science and Technology, Singapore School of Social Sciences, Singapore 599489, Singapore; 4 School of Medicine, Faculty of Health and Medical Sciences, Taylor’s University, Subang Jaya 47500, Malaysia

**Keywords:** sleep stages, classification, deep learning, CNNs, polysomnography (PSG)

## Abstract

Sleep disorder is a symptom of many neurological diseases that may significantly affect the quality of daily life. Traditional methods are time-consuming and involve the manual scoring of polysomnogram (PSG) signals obtained in a laboratory environment. However, the automated monitoring of sleep stages can help detect neurological disorders accurately as well. In this study, a flexible deep learning model is proposed using raw PSG signals. A one-dimensional convolutional neural network (1D-CNN) is developed using electroencephalogram (EEG) and electrooculogram (EOG) signals for the classification of sleep stages. The performance of the system is evaluated using two public databases (sleep-edf and sleep-edfx). The developed model yielded the highest accuracies of 98.06%, 94.64%, 92.36%, 91.22%, and 91.00% for two to six sleep classes, respectively, using the sleep-edf database. Further, the proposed model obtained the highest accuracies of 97.62%, 94.34%, 92.33%, 90.98%, and 89.54%, respectively for the same two to six sleep classes using the sleep-edfx dataset. The developed deep learning model is ready for clinical usage, and can be tested with big PSG data.

## 1. Introduction

Sleep disorders are widespread in most of the population, and may lead to serious health problems affecting the quality of life [1]. Insomnia, hypersomnias, parasomnias, sleep-related breathing, narcolepsy, circadian rhythm disorders, and sleep-related movement disorders are the common health problems that are created due to sleep disorders. Although many of these disorders can be diagnosed clinically, some of them should be analyzed using advanced techniques in the laboratory environment [1,2]. Polysomnogram (PSG) recordings of subjects are the physiological signals that are collected during an entire night of sleep. The PSG is a multivariate system consisting of signal recordings such as electroencephalogram (EEG), electrocardiogram (ECG), electrooculogram (EOG), and electromyogram (EMG) [3]. After the recordings, sleep stage scoring is performed on PSG records. This process is manually carried out by the sleep experts who score and grade the sleep stages [4]. These experts visually evaluate the PSG signals for a specific time frame, and then determine the scores according to various criteria. The main criteria for this process are based on the guidelines that were first proposed by Rechtschaffen and Kales (R&K) [5], and later developed by the American Academy of Sleep Medicine (AASM) [6]. According to the rules of R&K, a sleep stage can be classified as wake (W), four non-rapid eye movement (NREM) stages (S1–S4), and rapid eye movement (REM). According to the AASM guidelines, the S3 and S4 stages are represented by a single class as slow-wave sleep (SWS). The wake sleep stage is defined as the class of awakening of the subject before the sleep. NREM S1 is the first stage of a sleep where the brain activity slows down, and muscles are relaxed. Stage S2 is the stage where the actual sleep phase begins, and the eye movements stop in this stage. Stage S3 is called deep sleep, because the brain function of the subject is significantly reduced. Deep sleep activity continues in the NREM S4 sleep stage. Eyes are closed in the REM stage, but they also move rapidly [7].

The visual inspection of PSG signals and manual determination of sleep stages is a complex, costly, and problematic process that requires expertise [8,9]. Besides, it is visually hard to detect EEG signal variations due to their random and chaotic nature [10]. For this reason, automated detection and recognition systems are developed to assist the experts. The most commonly used PSG signal for sleep-stage classification is the EEG data of one or more channels. Usage of the EEG signal is mostly preferred because EEG signals can be easily obtained with wearable technologies, and they consist of useful information as well [10,11]. Wearable technologies are an important technological advancement because the usage of this technology helps monitor the sleep data of subjects comfortably in their home environment [12].

During EEG signal processing, feature extraction, feature selection, and classification [13] steps are commonly used. Time, frequency, time-frequency domain-based transformations, and non-linear feature extraction methods are employed by various researchers at the feature extraction stage of EEG signals [14,15,16]. Due to the characteristic features of these signals, more advanced signal-processing techniques and complex machine learning algorithms are preferred instead of time and frequency domain approaches [7,10,17,18,19]. However, all of these approaches are mostly based on the use of shallow classifiers on the features obtained from one or more handcrafted feature extraction/selection processes.

Acharya et al. [20] have performed the automatic identification of sleep stages with a Gaussian mixture model classifier using high-order spectra (HOS)-based features for two channels of EEG data. For the feature extraction stage, Sharma et al. [7] employed a novel three-band time-frequency localized wavelet filter bank, and then the extracted features were given as input to the support vector machine (SVM) classifier for the automated recognition of sleep-stages. Hassan et al. [21] first decomposed the EEG signals using ensemble empirical mode decomposition (EEMD), and then extracted several statistical properties from the data. For this purpose, they proposed a classifier called random undersampling boosting (RUSBoost), which can automatically score sleep with the obtained features. Zhu et al. [22] performed the sleep-stage recognition task with 14963 EEG segments using a graph domain-based approach. They mapped EEG signals into a visibility graph (VG) and a horizontal visibility graph (HVG). Rahman et al. [23] preferred discrete wavelet transform (DWT) for the feature extraction on single EOG signals, and they claimed the superiority of EOG signals over EEG signals in the classification of sleep stages. Tsinalis et al. [12] obtained sleep stage-specific signal characteristics using time-frequency-based feature extraction, and achieved an average accuracy of 86% on EEG data of 20 healthy young adults. Bajaj et al. [24] proposed an EEG-based technique that used time-frequency images (TFIs). Their method can automatically classify the data into sleep stages by using the least-square SVM classifier and the features from the histograms of segmented TFIs.

Huang et al. [9] used spectral feature extraction from two foreheads (FP1 and FP2) of EEG signals by using short-time fast Fourier transform and manual scoring knowledge. They also classified sleep stages with these features by using the relevant vector machine classification technique. Nakamura et al. [25] employed a multi-class SVM to classify the features derived from EEG by using multi-scale fuzzy entropy (MSFE) and multi-scale permutation entropy (MSPE) features. Similarly, Rodriguze-Sotelo et al. [4] used entropy-based features with an unsupervised classifier. Acharya et al. [26] proposed a solution for the recognition of six stages of sleep using non-linear parameters. Fell et al. [27] used a variety of spectral and non-linear measurements from EEG signals for the discrimination of sleep stages. They reported that the combinations of these measurements would produce better results than the previous studies, as indicated in the literature. In another study, Alickovic and Subasi [3] proposed a three-module structure for the same problem. In the first module of their solution, the signals obtained from the Pz–Oz channel were de-noised using multi-scale principal component analysis (PCA). In the second module, feature extraction was performed by using statistical methods on the signals separated to sub-bands by the DWT. Finally, in the third module, rotational SVM was used to classify the data into five-stage sleep data with an accuracy of 91.1%.

Imtiaz et al. [28] suggested a small decision tree (DT) driven by a class machine for the automated scoring of sleep stages. They reported 82% and 79% accuracy rates during training and testing, respectively. Silveria et al. [29] applied the DWT method on EEG signals and performed sleep-stage classification using the random forest (RF) classifier on kurtosis, skewness, and variances. Şen et al. [15] collected 41 attributes under four categories for the feature-extraction stage, and then used a variety of feature selection methods to select the useful features from these collected attributes. Memar and Faradji [30] also proposed a system for the classification of the wake and sleep stages. During the feature-extraction stage, they decomposed each EEG sample into eight sub-bands with different frequency contents, and then classified the extracted features using the random forest classifier. Yulita et al. [31] used a fast-convolutional method-based feature learning and softmax classifier for automatic sleep stage classification. Vural et al. [32] constructed an EEG-based classification structure using principal component analysis (PCA).

In the given state-of-the-art for the sleep stage classification methods, all of the feature extraction, selection, and classification tasks are performed on the data as separate processes. Recent developments in the field in the machine learning area have led to the emergence of end-to-end deep structures with the capability to perform these separated tasks together in a more efficient way [33,34,35,36]. Deep learning methods have already demonstrated their success in various research areas such as image recognition, sound processing, and natural language processing. Accordingly, deep models already have a widespread application in the biomedical area. There has been a notable increase in the use of deep learning approaches for the evaluation of biomedical signals (EEG, ECG, EMG, and EOG) [37]. Deep learning methodologies were employed on many challenging topics such as computer-based evaluations of ECG data for heart diseases [38,39,40,41] and the detection of neurological disorders using EEG signals [42,43,44,45]. There are also few studies in the literature where deep learning models have been used for the sleep stage classification. Supratak et al. [46] conducted a study on DeepSleepNet by combining a convolutional neural network (CNN) and bidirectional long short- term memory (BLSTM) for the sleep stage classification. DeepSleepNet contains a CNN and BLSTM as consecutive steps. The learning process was completed in the CNN part, and a sequence of residual learning was realized in the BLSTM part. Tsinalis et al. [12] categorized more than 20 healthy subject’s data by using a CNN model on single EEG channel data. They achieved an average performance of 74% accuracy for five-stage sleep classification. Tripathy and Acharya [47] classified sleep stages by using an RR-time series and EEG signals with a deep neural network (DNN). Chambon et al. [48] proposed an 11-layer two-dimensional (2D) CNN model for sleep stage classification. In this model, EEG/EOG and EMG PSG signals are used as the input. They reported that the usage of limited EEG channels (six channels) on their model showed similar performances with the use of 20 channels of EEG data. Michielli et al. [49] proposed a cascaded LSTM architecture for automated sleep stage classification using single-channel EEG signals.

In this study, a new deep learning model based on a one-dimensional convolutional neural network (1D-CNN) is proposed for automated sleep stage classification. This model helps construct an end-to-end structure where no handcrafted feature is used for sleep stage recognition with raw PSG signals. One of the most important contributions of the study is that the proposed deep model can be used without changing any of its layer parameters for two to six sleep classes and other types of PSG signals. Hence, our model is flexible and developed using two popular sleep databases that are available in the literature.

## 2. Materials and Methods

This section presents detailed information about the architecture of the proposed 1D-CNN model, and also provides information about the sleep datasets used in the study.

### 2.1. Sleep Datasets

In this study, the two most common public sleep datasets have been used to evaluate the proposed deep CNN model. The first of one is the sleep-edf [50] dataset, which contains eight healthy males and females’ polysomnograms (PSG) records. These PSG recordings include two EEG channels: one horizontal EOG signal (Fpz–Cz) and one submental chin EMG signal (Pz–Oz). These signals were obtained with a sampling rate of 100 Hz, and each 30-s fragment was scored based on the R&K manual. In the dataset, there are also hypnogram files, which contain annotations of sleep patterns for each subject according to PSGs. These patterns are labeled for sleep stages as W, S1, S2, S3, S4, REM, M, and ? (not scored). The second dataset, sleep-edfx [51], is the extended version of the sleep-edf database. The sleep-edfx dataset contains the PSG records of 61 subjects. In this work, *.edf records are divided into two different groups: Sc* and St*. The Sc* records include PSG records of a 24-hour routine of the subjects, and the St* records include one-night data from the hospital collected with a miniature telemetry system. Each dataset contains scored recordings for six sleep stages. Table 1 shows the detailed information of the used sleep records. It may be noted that no filtering was performed on the data, which is the significant difference compared to the other reported studies. Only ambiguous recordings are filtered out so that the number of records used is higher than the rest of the studies using this dataset.

**Figure 1:**
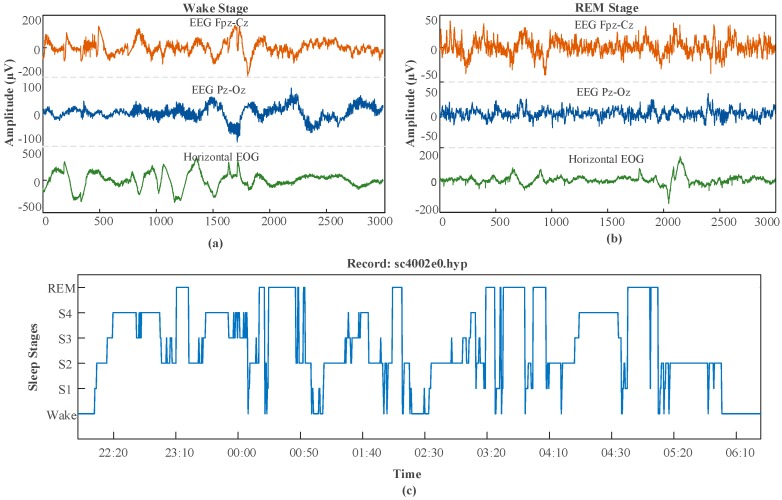
Sample polysomnogram (PSG) signals and scored hypnogram records obtained from the sleep-edf database: (**a**) wake stage, (**b**) rapid eye movement (REM) stage, and (**c**) hypnogram for sc40020 record between 22:20 and 06:10.

In both datasets, more than 50% of the records belong to wake (W) sleep stages, and the second highest number of records belongs to the class S1. In Figure 1, one segment of the PSG signal sample of the sc4002e0 record is shown for two stages (wake stage and rapid eye movement-REM stage). This record is chosen from the sleep-edf dataset, and the figure shows the hypnogram recording of the same (sc4002e0) record for the time of 22:20–06:10.

### 2.2. Deep Model Architecture

CNN models are frequently used to recognize the two-dimensional images [52]. However, the usage of CNN models is not limited to two-dimensional or three-dimensional recognition tasks. 1D-CNN shares the same properties with other CNN models. The only difference is in the convolution operation, which is called as the 1D convolution operation, and is known to be suitable for input data in one dimension, such as biomedical signals [37]. For a 1D input signal, *S*, and kernel *W*, the convolution operation is defined as follows:

(1) $${\left(S*W\right)}_{n}=\sum_{i=1}^{\left|W\right|}W(i)S(i+n-1)$$

In this equation, the ∗ operator denotes the discrete convolution operation. It is to be noted that the kernel, which is also called weights, slides over the input. The output of the convolution process is called a feature map. Let ${\left(S_{|}{}_{{}_{W(i,j)}}\right)}_{n}$ be the restricted matrix of the input matrix to the weight matrix. The elements of ${\left(S_{|}{}_{{}_{W(i,j)}}\right)}_{n}$ represent the elements of *S* from *n* to the dimension of *W*(*i*,*j*). Thus, the output matrix can be represented by a general formula, which is given in Equation (2):

(2) $$O_{n}^{l}={\left(S_{|}{}_{{}_{W(i,j)}}*W(i,j)\right)}_{n}$$

**Figure 2:**
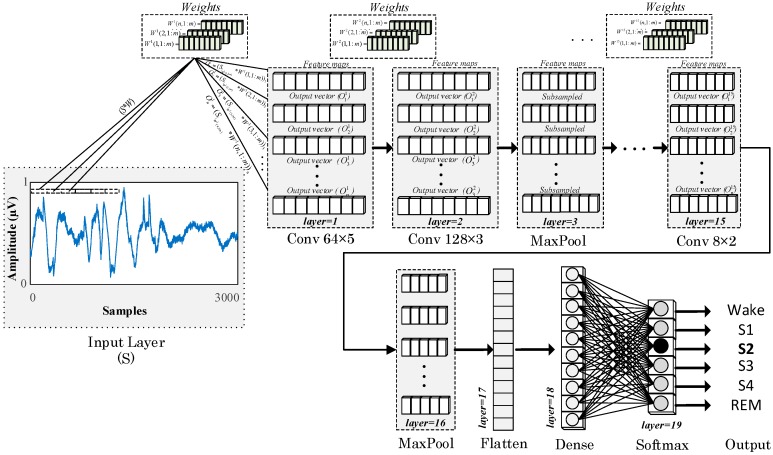
Block representation of the proposed one-dimensional convolutional neural network (1D-CNN) model for the classification of sleep stages.

This convolution layer operation is similar to the feature extraction stage, and its output produces a feature map of the input. Either feature maps can be sub-sampled into a pooling layer that is placed inside the model, or they can be processed in consecutive convolution layers. The final layer of the CNN model usually contains a neural network layer, which is called a fully connected layer, and this layer performs the classification task. Figure 2 presents the block diagram of the proposed 19-layered 1D-CNN model for the automated recognition of sleep stages.

The preprocessed PSG signal segments with 3000 samples are used as input. These PSG signals are convoluted in the first layer of the model with 64 × 5 filters and three stride ratios to produce feature maps in 64 × 999 sizes. The second layer of the model is another convolution layer with 128 × 5 filters. This layer generates new feature maps in 128 × 997 sizes by using the output of the previous layer. In the MaxPool layer, the maximum values in two unit regions of the two output vectors are reduced to a single value. Thus, the input feature maps are reduced to 128 × 498 dimensions. In the consecutive layers of the model, these processes are repeated in a similar fashion, but with filters of different sizes. Dropout layers are placed in the model to prevent the overfitting problem. The dimensions of the input vectors in the flattened layer are converted to the appropriate dimensions for the dense layers. Finally, in the softmax layer, the input signals are mapped to the output signals. Therefore, the number of units in this layer is the same as the number of classes (nb_class). All of the layers of the model and detailed parameter representations of these layers are given in Table 2.

The brute force technique is used to adjust the parameters and determine the number of layers in the model. The model’s validation performance curve is continuously adjusted by changing the parameters. During these operations, the PSG signals and data partitioning ratios are changed to obtain a single model, which would give the optimum result for each signal. For the model construction, only three PSG signals and six classes of the sleep-edf database are used. The rest of the sleep-edf classes (five, four, three, and two) and the whole sleep-edfx database are not used during the model construction. In addition, both the sleep-edf and sleep-edfx databases are divided into 70% training, 15% validation, and 15% test sets. The performance of the model is evaluated on unseen test sets.

## 3. Results

The experiments were carried out on the widely used sleep-edf and sleep-edfx datasets. The records in the dataset were converted into five classes; then, comprehensive evaluations were performed on the data. In this section, detailed information on the experimental setup and results are presented.

### 3.1. Experimental Setups

Raw PSG signals in the database were divided into 30-s segments. Thus, PSG segments with 3000 samples were used for each hypnogram value. In the experimental studies, the results were evaluated using different PSG signals taken from both sleep databases. We have analyzed the database using the following combinations of PSG signals: single-channel EOG, single-channel EEG, and single-channel EOG + single-channel EEG. Single-channel EOG channel signals contain one horizontal EOG signal in the recordings. The single EEG consists of the Fpz–Cz channel signal proposed in the literature [12,46,53] for each record. For the single-channel EOG + single-channel EEG, one horizontal EOG and Fpz–Cz EEG signal was used for each recording. When these signals were given as input to the CNN model, sample elimination was performed for the ambiguous scores (‘X’, ‘?’, so on). Additionally, no more filtering operations were performed, such as removing noisy signals from a certain range of amplitude. The preprocessing operations on signals contain the standardization and the normalization of signals to the zero to one range. The entire dataset was divided into three parts of 70%, 15%, and 15% for training, validation, and testing, respectively. Training and validation datasets were used to determine the layer parameters during the training phase of the model. The testing data was a new dataset that the model had not used or seen before. The test performance of the model was carried out on the trained model.

**Figure 3:**
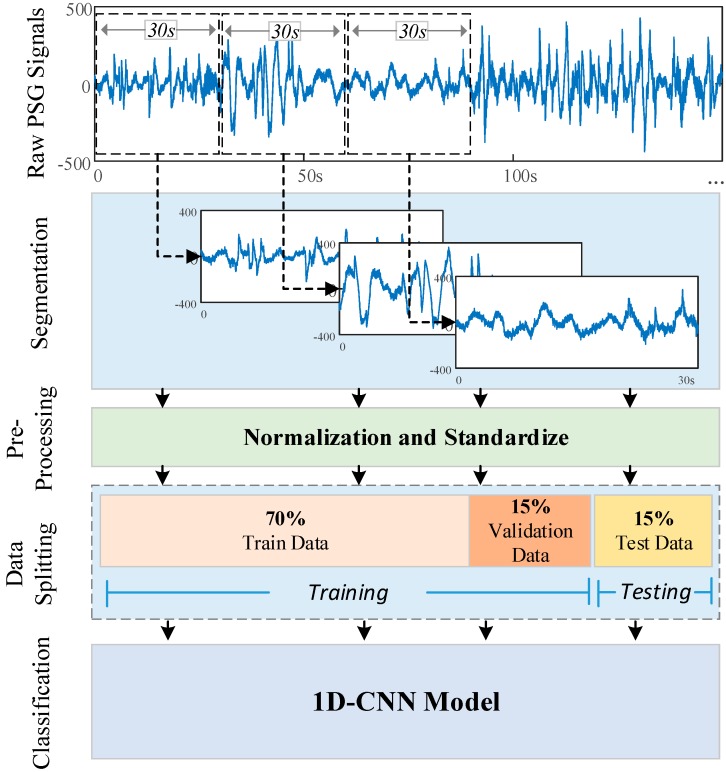
An illustration of experimental steps.

In order to ensure the consistency of the results, the random seed values were kept constant in the data-splitting processes. For all the experimental results, the training phase of the 1D-CNN model was carried out for 100 epochs, and the same hyperparameters of the model were used for all of the datasets. These hyperparameters are as follows: the Adam optimizer, the learning rate was 0.0001, and the decay was 0.003. Figure 3 illustrates the experimental steps.

Deep learning implementation was realized by using the Python programming language. Keras was used to create the model and collect the experimental results, and Tensorflow was used as the backend. For testing, a computer with Intel Core i7-7700HQ 2.81GHz CPU, 16 GB RAM, and a NVIDIA GeForce GTX 1070 8 GB graphics card was used. Experimental results were obtained for five different sleep classes. Definitions of these classes are given in Table 3.

### 3.2. Results

The experimental results were collected separately for the sleep-edf and sleep-edfx datasets. In addition to comprehensive evaluations with these datasets, further experimental results were presented according to PSG signals and two to six sleep classes. In these studies, we have used standard single-channel EEG and single channel EOG signals [22,23,32,54].

#### 3.2.1. Results of Sleep-edf Database

Sleep-stage estimations were performed on eight subject records of the sleep-edf database using different PSG signals. The PSG signals that were used in this database are, single-channel EOG (one horizontal EOG), single-channel EEG (Fpz–Cz channel), and single-channel EOG + single-channel EEG, which is a combination of the single-channel EOG and single-channel EEG, respectively. There are 15,188 samples in this database. The distribution of these samples for training, validation, and testing are 10,630 (70%), 2279 (15%), and 2279 (15%), respectively.

##### Using Single EOG Signal 

**Figure 4:**
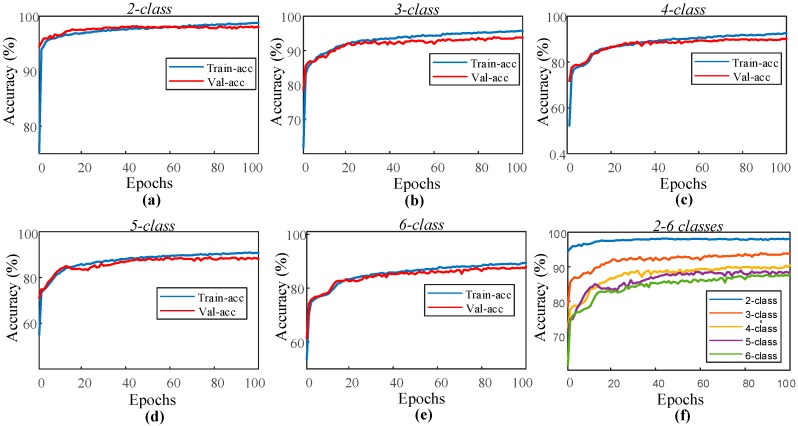
Performance graphs of the proposed 1D-CNN model with the single EOG signal for the sleep-edf dataset: (**a**) two-class model, (**b**) three-class model, (**c**) four-class model, (**d**) five-class model, (**e**) six-class model, and (**f**) validation accuracy for the models with two to six classes.

In the sleep-edf dataset experiment, the proposed 1D-CNN model was trained for the automated recognition of sleep stages by only using the EOG channel of the subjects. The layer parameters and hyperparameters of the model remained unchanged for all two to six classes. Figure 4 shows the performance graphs of the proposed 1D-CNN model during the training of each class.

As can be seen from the performance graphs, no overfitting problem occurred for all of the classes. The training and validation accuracy curves indicate positive learning. The highest recognition for the performance was observed for the second class, and the lowest performance was received with the sixth class. The model for which each class is individually trained has been applied for the test data of these classes. These test sets consist of data that the model has never seen before. Table 4 presents the accuracy values of the 1D-CNN model for each class in the training and testing stages.

**Figure 5:**
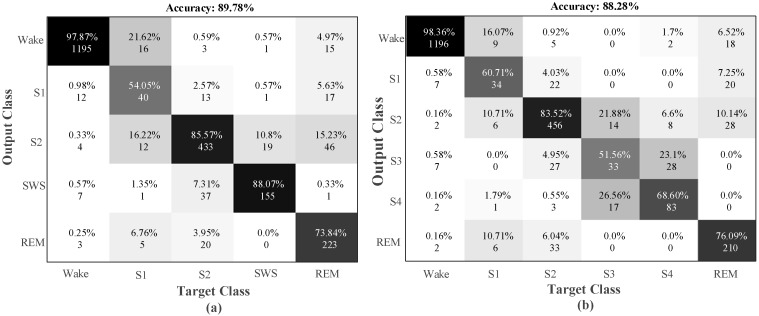
Confusion matrix obtained using single-channel EOG input signals for the sleep-edf data for: (**a**) five-class test data and (**b**) six-class test data.

The testing data had never been used during training. As can be seen in Table 4, the training and testing accuracy values are close to each other. Hence, it implies that the proposed model has good generalization ability. It can be seen from Table 4 that the highest recognition performance for both the training and testing phase was obtained for two classes, which contained two sleep stage classes. The model has provided 98.06% accuracy on the test data for this class. The lowest recognition rates were obtained for the six-class and five-class datasets. Figure 5 shows the confusion matrixes obtained for six-class and five-class test data.

The values in the confusion matrix cells are the precision ratio and the number of samples. The values in the diagonal line represent the stages that are correctly recognized, and the values outside the diagonal region represent the incorrect recognition. For example, 1196 of the 1230 test data of the wake (W) stage were correctly recognized, while 34 were incorrectly classified into other stages. In Table 5, various performance evaluation parameters obtained from 2550 of the test data values of these classes are presented.

A sensitivity of 0.97 was obtained for the wake sleep stage. Since the majority of data belong to this stage, the learning model showed a trend toward learning the data in this stage. The lowest rate of sensitivity was observed in the stage S1 as 0.48. The amount of data in stage S1 is less than others, so that the proposed model had difficulty in learning this stage. The five-class and six-class models achieved 89.78% and 88.28% accuracy rates, respectively.

##### Using Single-Channel EEG Signal

**Figure 6:**
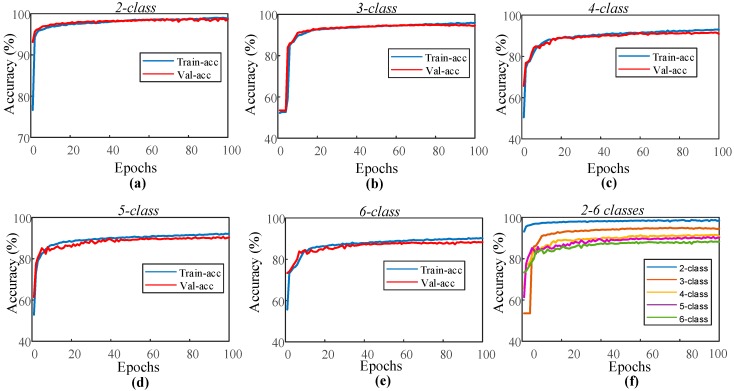
Performance graphs of the proposed 1D-CNN model with the single channel EOG signal for the sleep-edf dataset: (**a**) two-class model, (**b**) three-class model, (**c**) four-class model, (**d**) five-class model, (**e**) six-class model, and (**f**) validation accuracy for the models with two to six classes.

Few studies in the literature have obtained results using only one EEG channel from the PSG signals. For this reason, performance evaluation of the model using the EEG signal of the Fpz–Cz channel is given for the sleep-edf data. The parameters and data (training, validation, and test) of the 1D-CNN model for the single EOG signal remained unchanged, and the model was applied to the EEG signal. Figure 6 presents the training and validation accuracy graphs of the proposed model for two to six classes using the sleep-edf dataset. Compared to similar graphs obtained in Figure 4 for the single-channel EOG, it can be seen that the training and validation curves in Figure 6 are close to each other. The proposed model for this PSG signal did not show any overfitting or underfitting problems during training, and the training performance is higher than the one obtained using EOG signals. Table 6 shows the performance values of the model for training and testing with single-channel EEG signals.

**Figure 7:**
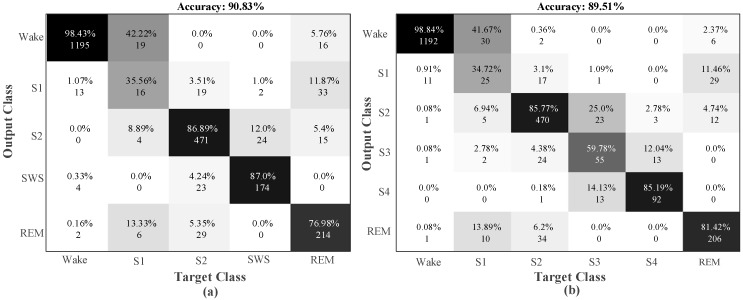
Confusion matrix obtained for single-channel EEG input signals of the sleep-edf data: (**a**) five-class test data and (**b**) six-class test data.

For single-channel EEG signals, the model has captured the highest test accuracy rate as 98.33% for the two-class model, which consisted of only two sleep stages. The accuracy rate for the five-class model was 90.83%, and for the six-class model, it was 89.51%. Figure 7 shows the confusion matrixes obtained from the test data of the five-class and six-class datasets.

For wake stage, 98.4% accuracy was obtained for the five-class model, and 98.8% accuracy was obtained for the six-class model. In stage S1, 35.6% and 34.7% accuracy rates were observed for the five-class and six-class models, respectively. The accuracy rate for the five-class model of the REM stage was 4.4% lower than the six-class model. The detailed values of the evaluation parameters for these classes are given in Table 7.

##### Using Single EOG + EEG Signals

The final experiment with the sleep-edf database contained a combination of EOG and EEG signals, unlike the other studies in the literature. For this purpose, the EEG and Fpz–Cz channel EEG signals from each instance of PSG signals were chosen as the inputs to the proposed model.

**Figure 8:**
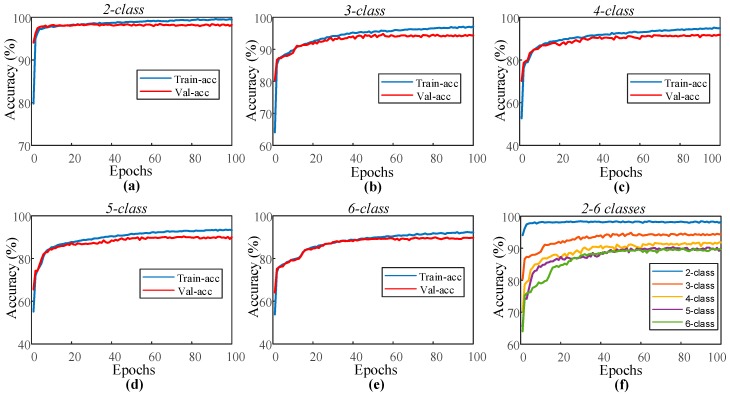
Performance graphs of the proposed 1D-CNN model with a single-channel EOG+EEG signal for the sleep-edf dataset: (**a**) two-class model, (**b**) three-class model, (**c**) four-class model, (**d**) five-class model, (**e**) six-class model, and (**f**) validation accuracy for the models with two to six classes.

For these PSG signals, the performance of the model during the training phase increased compared to the other signals (see Figure 8). There was a noticeable increase in the training performance of the model, so the difference between the training and validation curves increased. Table 8 shows the training and testing performance values of the proposed model for all of the classes using the EOG + EEG input signals. The accuracy value for the five-class test data was increased to 91.22%. For the six-class test data, less than 90% accuracy was observed with the previous signals, but here, it increased to 91.00% with PSG signals. The main reason for this may be the use of two different PSG signals in the input layer of the model, which lead to more distinctive features for the classes.

#### 3.2.2. Results on Sleep-edfx Database

Sleep-edfx, which is an extended version of the sleep-edf database, contains sleep data for 61 subjects. Experimental studies were carried out for three separate signals as single-channel EEG, single-channel EEG, and single-channel EEG + EOG using sleep-edfx data. The sleep-edfx database contains 127,512 samples. In the experimental studies, 70% (89,258) of the data was used for training, 15% (approximately 19,127) of the data was used for validation, and the remaining 15% (19,127) of the data was used for testing.

##### Single-Channel EOG

**Figure 9:**
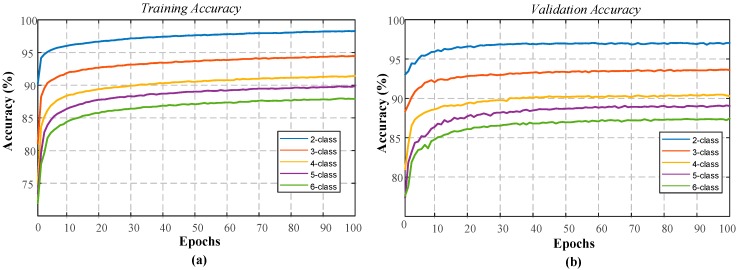
Training performance graphs obtained for different classes when the proposed model used the single-channel EOG from the sleep-edfx database: (**a**) training accuracy and (**b**) validation accuracy.

Training and testing of the 1D-CNN model were carried out using only one horizontal EOG channel belonging to 61 instances. The same model layer parameters (used for the previous database) were used without any changes for this database also. Figure 9 shows the performance graphs of the proposed model obtained during the training phase with the EOG signal.

The proposed model has obtained the highest training and validation accuracy values for the two-class experiments. The training and validation performances of the model for all the two-class, three-class, and four-class experiments were obtained over 90%. The detailed numerical values of the experiments are given in Table 9.

**Figure 10:**
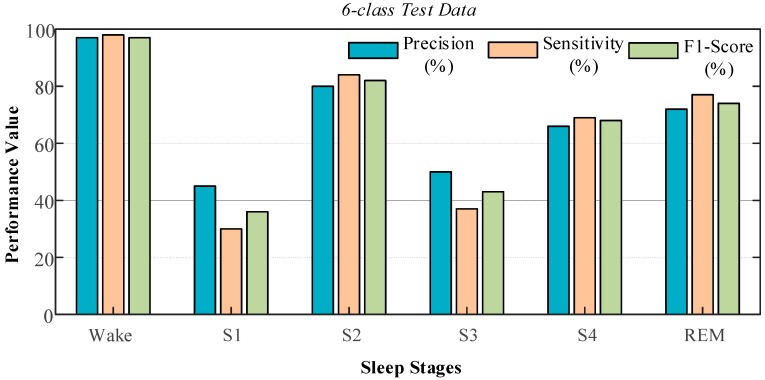
Graphs of performances obtained using the six-class testing data.

The trained model achieved 97.13% performance for the two-class dataset when fed with the unseen data. The lowest test performance of 87.08% was obtained for the six-class dataset, which contained the six different sleep stages. For this class, the values of the performance criteria were obtained using the test data, which is given in Figure 10, in detail.

The highest values of the evaluation performance were obtained for the wake stage where the precision, sensitivity, and F1-score values were 97%, 98%, and 97% respectively. The S1 and S3 stages produced the lowest performance values. The precision value for stage S1 was 45%, and the sensitivity value for this stage was 30%. For the S3 stage, these values were calculated as 50% and 37%, respectively. The most critical factor for these low performance scores was the imbalance in the distribution of data in each stage. For example, there is a big difference in the distribution of the test data for the wake, S1, and S2 stages. In the test data, 11,257 samples belonged to the wake stage, while the data belonging to stages S1 and S2 included 707 and 799 samples, respectively.

##### Single-Channel EEG

**Figure 11:**
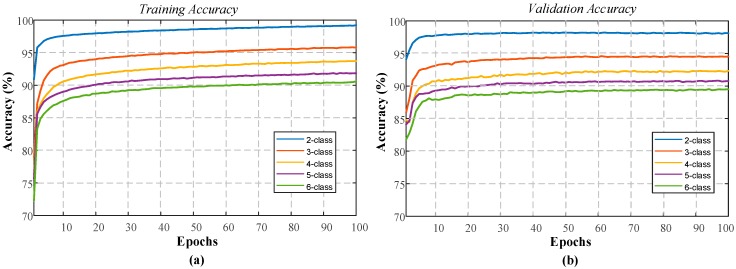
Performance graphs obtained for the proposed model using sleep-edfx database with single-channel EEG: (**a**) training accuracy, and (**b**) validation accuracy.

The final experimental study for the sleep-edfx database was the use of the single-channel EEG signal (of the Fpz–Cz channel from the PSG signals). The single-channel EEG data was used to train the model. Figure 11 shows the performance graphs of the 1D-CNN model presented for a 100 epoch period. These graphs were generated for the training phase of all the classes using the single EEG database.

When the performance graphs of the single-channel EEG experiments were examined, performance improvement could be observed for both the training and validation phases. The training performance for the two-class model reached 99.21%, while the validation accuracy increased to 98.09%. Similarly, an increase of around 2% was observed for the other classes. Table 10 shows the training and testing performances of the proposed model using the sleep-edfx database with a single EEG signal.

**Figure 12:**
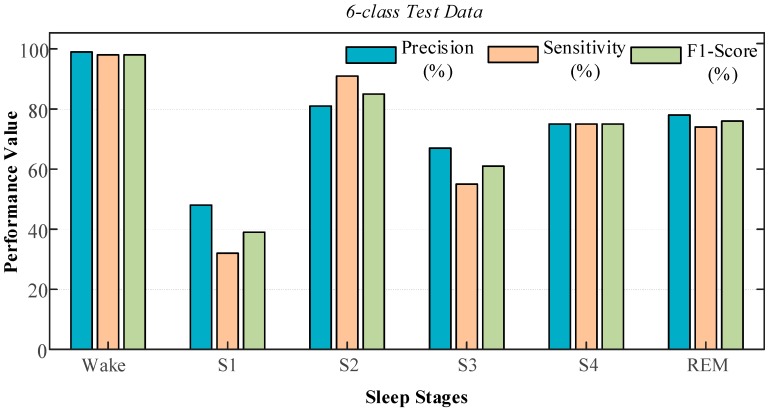
Graphs of performance measures for the six-class model during the testing phase with single-channel EEG signals obtained from the sleep-edfx database.

It can be seen from the test performances that the trained model showed a performance of more than 90% in all the other sets, except for the six-class set (Table 10). The accuracy rate of 87.08% with a single-channel EOG increased to 89.43% with the use of the EEG signal for the six-class dataset. Figure 12 shows the precision, sensitivity, and F1-score graphs belonging to the various sleep classes during testing class for the six-class dataset. Values of 99% precision and 98% sensitivity were obtained for the wake stage during the testing phase with single-channel EEG input. We have obtained 48% precision and 32% sensitivity for stage S1. The obtained results with this data are better than the ones using single-channel EOG data.

#### 3.2.3. Summary of Results

In this study, two popular datasets—sleep-edf, and sleep-edfx—were used. Five different sleep classes (two to six) were created from these datasets. Comprehensive experimental results were presented using different PSG signals. In all of the experimental studies, only a single model was used without any change in the layer parameters. Thus, an effective end-to-end model was created without any manual feature extraction or selection procedure for sleep scoring. Table 11 presents the summary of results that was obtained for the various combinations of the used data and the sleep stage using the same CNN model.

The highest recognition rate of 98.33% was obtained for the two-class (C = 2) dataset using single-channel EEG signals with the sleep-edf dataset. For the rest of the classes, the highest results were obtained when the EEG and EOG signals were used together. A recognition performance of 91.00% was achieved for the dataset with six classes. The highest accuracy for each class was obtained using the sleep-edfx database with the use of single-channel EEG data. The highest recognition rate for the six-class stages in this database was 89.43%.

## 4. Discussion

Many studies have been conducted on the sleep stage classification using sleep-edf and sleep-edfx datasets using machine-learning techniques, and so far, very few of them have used deep learning models. We have summarized these studies in two different tables. Table 12 presents the comparison of various studies carried out on the classification of sleep stages using sleep-edfx data.

In this study, we have used 127,512 samples of sleep-stage signals obtained from the sleep-edfx dataset. Our accuracies that were obtained by using single-channel EEG + EOG data for two to six sleep classes were 97.62%, 94.34%, 92.33%, 90.98%, and 89.54%, respectively. The accuracy rates that were obtained using EEG + EOG signals were marginally better than the single-channel EEG and single-channel EOG signals.

Table 13 presents a summary of the automated classification of sleep stages using the sleep-edf database. In this study, we used 15,188 samples of sleep-stage signals from the sleep-edfx dataset. Our accuracies that were obtained by using single-channel EEG data for two to six sleep classes were 98.06%, 94.64%, 92.36%, 91.22%, and 91.00%, respectively. The accuracy rates that were obtained using EEG signals were marginally better than the EOG signals.

As can be seen from the comparison tables (Table 12 and Table 13) that the number of sleep-edfx dataset samples differed from those of previous studies. There is no detailed information in these studies about this data elimination. Differently, we have used the highest number of samples for this database (127,512). Moreover, few of these methods have been evaluated only for five or six classes. The results show that the proposed method provided excellent performance for two to six classes on both the datasets. In addition, most of the other studies used handcrafted feature extraction/selection operations and shallow classifiers. From this perspective, the proposed model differs from the rest, and provides good potential as a complete end-to-end structure.

The advantages of the proposed study are given below:

- The proposed model is tested using two popular datasets for two to six sleep classes.
- With a single model, without changing any layer or hyperparameter values, the detection performance is significantly improved for five classes (two to six sleep classes) using different PSG signals of both datasets.
- A complete end-to-end recognition structure is developed without the need for any manual feature extraction stages.

The main disadvantages of the proposed classification system are as follows:

- Performance regarding distinguishing a few stages (especially S1) needs to be improved. One solution could be the use of more data in each stage.
- Elimination of noise from the PSG signals is a challenging task.

In future studies, the proposed model will be tested using different sleep databases. In this study, we have evaluated the performance of the model based on signal-level criteria. We will use patient-level criteria to evaluate the performances of the model for the future works. Furthermore, it increasing the recognition performance by using different deep learning approaches such as a combination of CNN and LSTM can be a further aim.

## 5. Conclusions

In this study, a 19-layer 1D-CNN model is proposed for the classification of sleep stages. This model has an end-to-end complete architecture, and does not require separated feature extraction/selection and classification stages. The application of this model produced high recognition performance on different sleep classes when popular PSG input signals such as EEG and EOG are used. For the evaluations, two popular public databases for the automated sleep stage classification are used. In the sleep-edf database, there are 15188 samples of six sleep stages. The second database sleep-edfx contains 127512 samples. One major difference between this study and previous studies is the number of used samples, which has not decreased too much with the preprocessing steps. Performance comparisons using single-channel EEG, single-channel EOG, and a combination of EEG and EOG signals are given. These results revealed that the proposed model achieved the highest classification accuracies for two to six sleep classes as 98.06%, 94.64%, 92.36%, 91.22%, and 91.00% respectively. In the future, the model will be employed on the detection of other sleep-related abnormalities. The development of such fully automatic recognition systems could replace the task of traditional error-prone manual expert inspection of large-scale PSG signals.

## Figures and Tables

**Table 1:** Detailed information about the sleep database records used in this study.

Database	Sleep Stages						Total Samples
	Wake (W)	S1	S2	S3	S4	REM (R)	
**sleep-edf**	8055 (53.03%)	604 (3.97%)	3621 (23.84%)	672 (4.42%)	627 (4.12%)	1609 (10.59%)	15,188
**sleep-edfx**	74,676 (58.56%)	4848 (3.8%)	27,292 (21.4%)	5075 (3.9%)	3773 (2.9%)	11,848 (9.2%)	127,512

**Table 2:** Details of layers and parameters used in the proposed 1D-CNN model.

Num.	Layer Name	No. of Filter × Kernel Size	Region/Unit Size	Layer Parameters	No. of Trainable Parameters	Output Size
1	1D Conv	64 × 5	-	ReLU, Stride = 3	384	64 × 999
2	1D Conv	128 × 5	-	ReLU, Stride = 1	24,704	128 × 997
3	MaxPool	-	2	Stride = 2	0	128 × 498
4	Dropout	-	-	Rate = 0.2	0	128 × 498
5	1D Conv	128 × 13	-	ReLU, Stride = 1	213,120	128 × 486
6	1D Conv	256 × 7	-	ReLU, Stride = 1	229,632	256 × 480
7	MaxPool	-	2	Stride = 2	0	256 × 240
8	1D Conv	256 × 7	-	ReLU, Stride = 1	262,272	128 × 233
9	1D Conv	64 × 4	-	ReLU, Stride = 1	32,832	64 × 230
10	MaxPool	-	2	Stride = 2	0	64 × 115
11	1D Conv	32 × 3	-	ReLU, Stride = 1	6176	32 × 113
12	1D Conv	64 × 6	-	ReLU, Stride = 1	12,352	64 × 108
13	MaxPool	-	2	Stride = 2	0	64 × 54
14	1D Conv	8 × 5	-	ReLU, Stride = 1	2568	8 × 50
15	1D Conv	8 × 2	-	ReLU, Stride = 1	136	8 × 49
16	MaxPool	-	2	Stride = 2	0	8 × 24
17	Flatten	-	-	-	0	1 × 192
18	Dense	-	64	ReLU, Drop = 0.2	12,352	1 × 64
19	Dense	-	nb_class	Softmax	195	1 × nb_class

**Table 3:** Notations used for sleep classes in this work.

Sleep Classes (C)	Sleep Stages
6	Wake―S1―S2―S3―S4―REM
5	Wake―S1―S2―SWS{S3 + S4}―REM
4	Wake―{S1 + S2}―SWS{S3 + S4}―REM
3	Wake―{S1 + S2 + S3 + S4}―REM
2	Wake―Sleep {S1 + S2 + S3 + S4 + REM}

**Table 4:** Performance values for sleep-edf data of the model for two to six classes using a single electrooculogram (EOG) signal.

Sleep Classes (C)	Model Accuracy Rate (%)		
	Training	Validation	Testing
2	98.87	98.02	98.06
3	95.66	93.76	93.76
4	92.48	90.38	91.88
5	90.76	88.14	89.78
6	89.39	87.84	88.28

**Table 5:** Various performance values obtained for five-class and six-class test data using a sleep-edf database with single-channel EOG.

Classes	Sleep Stages	Precision	Sensitivity	F1-Score	Number of Data
5-class	Wake	0.98	0.97	0.98	1230
	S1	0.54	0.48	0.51	83
	S2	0.86	0.84	0.85	514
	SWS	0.88	0.77	0.82	201
	REM	0.74	0.89	0.81	251
6-class	Wake	0.98	0.97	0.98	1230
	S1	0.61	0.41	0.49	83
	S2	0.84	0.89	0.86	514
	S3	0.52	0.35	0.42	96
	S4	0.69	0.78	0.73	106
	REM	0.76	0.84	0.80	251

**Table 6:** Performance values for the sleep-edf data of the model for two to six sleep classes using a single electroencephalogram (EEG).

Sleep Classes (C)	Model Accuracy Rate (%)		
	Training	Validation	Testing
2	98.93	98.63	98.33
3	96.03	94.60	94.20
4	92.92	90.86	91.39
5	92.07	90.25	90.83
6	90.01	88.32	89.51

**Table 7:** The performance values obtained for the five-class and six-class test data using the sleep-edf dataset with a single-channel EEG signal. SWS: slow-wave sleep.

Classes	Sleep Stages	Precision	Sensitivity	F1-Score	Amount of Data
5-class	Wake	0.98	0.97	0.98	1230
	S1	0.36	0.19	0.25	83
	S2	0.87	0.92	0.89	514
	SWS	0.87	0.87	0.87	201
	REM	0.77	0.85	0.81	251
6-class	Wake	0.99	0.97	0.98	1230
	S1	0.35	0.30	0.32	83
	S2	0.86	0.91	0.89	514
	S3	0.60	0.58	0.59	95
	S4	0.85	0.87	0.86	106
	REM	0.81	0.82	0.82	251

**Table 8:** Performance values using sleep-edf data of the models featuring two to six-classes of sleep stages with a single-channel EEG + EOG signal.

Sleep Classes (C)	Model Accuracy Rate (%)		
	Training	Validation	Testing
2	99.41	98.24	98.06
3	97.19	94.29	94.64
4	94.89	91.79	92.36
5	93.27	90.03	91.22
6	92.22	89.72	91.00

**Table 9:** Performance values for the sleep-edfx data of the models featuring two to six classes using single-channel EOG.

Sleep Classes (C)	Model Accuracy Rate (%)		
	Training	Validation	Testing
2	98.28	97.03	97.13
3	94.45	93.60	93.35
4	91.42	90.29	90.19
5	89.86	89.02	88.75
6	87.79	87.43	87.08

**Table 10:** Performance values achieved by the proposed models for two to six classes using the sleep-edfx database with a single-channel EEG signal.

Sleep Classes (C)	Model Accuracy Rates (%)		
	Training	Validation	Testing
2	99.21	98.09	97.85
3	95.77	94.52	94.23
4	93.72	92.33	92.24
5	91.85	90.75	90.48
6	90.59	89.50	89.43

**Table 11:** Summary of results obtained for various combinations of data used and sleep stages.

Database	PSG Signals	Accuracy Rates (%)				
		Sleep Classes (C)				
		C = 2	C = 3	C = 4	C = 5	C = 6
Sleep-edf	Single-channel EOG	98.06	93.76	91.88	89.77	88.28
	Single-channel EEG	98.33	94.20	91.39	90.82	89.51
	Single-channel EEG + EOG	98.06	94.64	92.36	91.22	91.00
Sleep-edfx	Single-channel EOG	97.13	93.35	90.19	88.75	87.08
	Single-channel EEG	97.85	94.23	92.24	90.48	89.43
	Single-channel EEG + EOG	97.62	94.34	92.33	90.98	89.54

**Table 12:** Summary of automated sleep stage classification using the sleep-edfx dataset. CNN: convolutional neural network, DWT: discrete wavelet transform, SVM: support vector machine.

Study	Number of Channel(s)/Signals	Number of Samples	Method	Accuracy Rates (%)				
				Sleep Classes (C)				
				C = 2	C = 3	C = 4	C = 5	C = 6
Ref. [25]	1 EEG	126,699	MSFE + MSP + SVM	-	-	-	93.8	-
Ref. [23]	1 EEG	54,587	DWT + SVM	-	-	-	-	91.7
Ref. [29]	1 EEG	106,376	DWT + RF	-	93.9	92.3	91.5	90.5
Ref. [28]	2 EEGs	59,316	SM + DT	-	-	-	78.85	-
Ref. [46]	1 EEG	41950	CNN + BLSTM	-	-	-	82.00	-
Proposed	1 EEG	127,512	1D-CNN	97.85	94.23	92.24	90.48	89.43
Proposed	1 EOG	127,512	1D-CNN	97.13	93.35	90.19	88.75	87.08
Proposed	1 EEG + 1 EOG	127,512	1D-CNN	97.62	94.34	92.33	90.98	89.54

**Table 13:** Summary of works conducted on the automated classification of sleep classes using the sleep-edf dataset.

Study	Number of Channel(s)/Signals	Number of Samples	Method	Accuracy Rates (%)				
				Sleep Classes (C)				
				C = 2	C = 3	C = 4	C = 5	C = 6
Ref. [25]	1 EEG	14,995	MSFE + MSP + SVM	-	-	-	93.8	-
Ref. [32]	2 EEGs	-	PCA	-	-	-	-	69.98
Ref. [21]	1 EEG	15,188	EEMD + RUSBoost	98.15	94.23	92.66	83.49	88.07
Ref. [55]	1 EEG	15,188	CEEMDAN + Bagging	99.48	94.10	92.14	90.69	86.89
Ref. [22]	1 EEG	14,963	(VG-HVG) + SVM	97.90	92.6	89.3	88.9	87.5
Ref. [23]	1 EEG	15,188	DWT + SVM	98.24	94.10	92.89	91.02	90.26
Ref. [23]	1 EOG	15,188	DWT + SVM	-	-	-	92.60	91.70
Ref. [7]	1 -EEG	15,139	Wavelet filter + SVM	97.8	93.5	90.7	89.9	89.5
Proposed	1 EEG	15,188	1D-CNN	98.33	94.20	91.39	90.82	89.51
Proposed	1 EOG	15,188	1D-CNN	98.06	93.76	91.88	89.77	88.28
Proposed	1 EEG + 1 EOG	15,188	1D-CNN	98.06	94.64	92.36	91.22	91.00
